# Snapshot Hyperspectral Volumetric Microscopy

**DOI:** 10.1038/srep24624

**Published:** 2016-04-22

**Authors:** Jiamin Wu, Bo Xiong, Xing Lin, Jijun He, Jinli Suo, Qionghai Dai

**Affiliations:** 1 Department of Automation, Tsinghua National Laboratory for Information Science and Technology (TNList), Tsinghua University, Beijing, 100084, China

## Abstract

The comprehensive analysis of biological specimens brings about the demand for capturing the spatial, temporal and spectral dimensions of visual information together. However, such high-dimensional video acquisition faces major challenges in developing large data throughput and effective multiplexing techniques. Here, we report the snapshot hyperspectral volumetric microscopy that computationally reconstructs hyperspectral profiles for high-resolution volumes of ~1000 *μm* × 1000 *μm* × 500 *μm* at video rate by a novel four-dimensional (4D) deconvolution algorithm. We validated the proposed approach with both numerical simulations for quantitative evaluation and various real experimental results on the prototype system. Different applications such as biological component analysis in bright field and spectral unmixing of multiple fluorescence are demonstrated. The experiments on moving fluorescent beads and GFP labelled drosophila larvae indicate the great potential of our method for observing multiple fluorescent markers in dynamic specimens.

With the rapid development of biological and materials science, more and more microscopic imaging applications are seeking to capture higher dimensional visual signals, such as high-speed volumetric data^1^,^2^ and hyperspectral data^3^, rather than traditional two-dimensional (2D) images. Developing high-speed hyperspectral volumetric microscopy can provide an effective tool for various applications such as multi-fluorescence labelled dynamic observation^4^, high-throughput chromatography^5^, medical science^6^ and material analysis under changing environment^7^.

However, such high-dimensional data raises high demands for data throughput. Although many methods have been proposed recently for high-speed volumetric imaging^1^,^8^ and snapshot hyperspectral imaging^9^,^10^, no one has ever realized high-speed hyperspectral volumetric imaging in optical microscopy, which needs to capture the five-dimensional (5D) data, i.e. three-dimensional (3D) spatial, one-dimensional (1D) spectral and 1D temporal dimensions. Most methods sacrifice the temporal resolution for additional dimensions such as axial dimension and spectral dimension. For example, confocal microscopy^11^, light sheet microscopy^12^ and existing hyperspectral microscopy^13^,^14^,^15^ still require large amounts of time for the extensive scanning of the whole 3D sample, point by point or line by line. The restriction caused by the acquisition speed makes it unable to capture dynamic changes of biological samples. To ensure the sufficiency of sampling the 5D data, instantaneous data throughput is an inevitable problem.

In addition, when we try to extend the traditional 2D images into higher dimensional information at high speed, obtaining high-dimensional sampling and high light efficiency are two main challenges with available devices. Thus novel multiplexing^3^ and coupled sampling^16^ methods should be designed. By scanning the 2D sensors, it is easy to achieve traditional 3D tomography with data multiplexing in spatial domain. Temporal dimension can be further introduced for 4D tomography utilizing the repeatability of the process^17^, which, in other words, can be viewed as the development of the 3D sensor for capturing the video of 3D volumes. But the 3D sensor is still unable to meet the requirement of high-speed hyperspectral volumetric data (5D data) acquisition. The 4D sensor with new multiplexing strategy, which can capture the 3D spatial information and additional 1D spectral information within a snapshot, is necessary to be introduced. Due to the rapid growing demands in various applications, it is challenging but essential to explore approaches for high-speed hyperspectral volumetric data capturing.

Here, we propose a novel approach for snapshot hyperspectral volumetric microscopy (SHVM), providing a new tool for recording the instantaneous 4D information of dynamic samples, which, to the best of our knowledge, has never appeared before. The first thing that crosses our mind is the combination of the absorptive micro-filter array^3^ and the microlens array^8^,^18^ for spatial-spectral coupled 4D sampling, but the data throughput of a single sensor is far from being sufficient to afford the 5D data. Therefore, we adopt camera array based light field microscopy^19^ for building the 4D sensor with the capability of large instantaneous data throughput (about 1778 MB/s for RGB sensors). To provide the 4D sampling with high light efficiency, data multiplexing is introduced by placing different broadband colour filters in front of the sensor array (see Fig. 1). Then each captured sensor image is the convolution of hyperspectral volumetric data with the corresponding spatial-spectral coupled 4D point spread function (PSF). A novel 4D deconvolution algorithm is also proposed to reconstruct the high-resolution hyperspectral volumetric data from the spatial-spectral coupled sampling without enforcing strong assumptions on the microscopic samples.

Fast volumetric imaging has been widely applied in biomedical studies^8^,^20^,^21^. The additional spectral dimension further facilitates various applications, including microscopic chromatography^5^, skin analysis^22^ and spectral unmixing of different dyes for fluorescence microscopy^14^. Both abilities are integrated in our system and provide a more powerful and flexible microscopy for new discoveries and comprehensive observation. Biological component analysis for 3D samples such as all kinds of materials and organisms becomes more convenient. Our system can work in different modes such as bright field microscopy and fluorescence microscopy to meet different requirements of applications. The experiment for volvox in bright field was conducted to show the potential application of 3D biological component analysis in object recognition. Further experiments on both static and dynamic fluorescent samples with our system were demonstrated to indicate its great potential in *in-vivo* 3D observation of multi-fluorescence labelled samples with spectrum unmixing^23^ and various studies including both morphological and spectral changes^6^.

## Results

### Experimental setup

**Figure 1:**
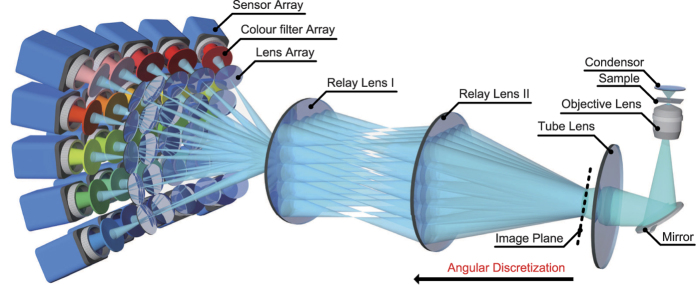
Schematic of the prototype system. The image plane of a microscope is relayed by two relay lenses to expand the aperture plane to the size of the lens array. Broadband colour filters with different spectral profiles are placed between the lens array and the sensor array for spatial-spectral coupled sampling. For 2D sensors physically layout in 2D array, they capture the information of the sample from different angles with the modulation of different colour filters (25 selected plane waves are shown in the figure).

**Figure 2:**
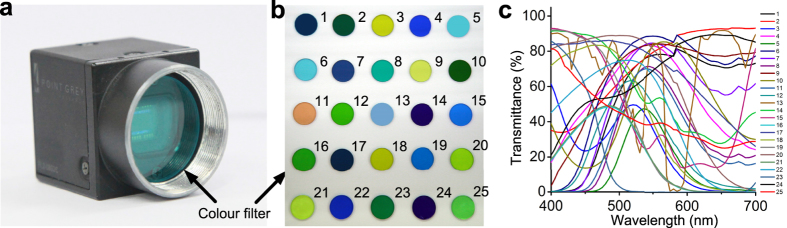
The properties of the colour filter array. (**a**) The position for each colour filter to be mounted. (**b**) The spatial layout of the colour filter array. (**c**) The spectral responses of the colour filters.

Figure 1 shows the schematic of the SHVM (more in Supplementary Fig. S1). Two different relay lens (Canon EF and Computar M0814-MP2) were used to expand the aperture plane of a commercial microscope (Olympus IX73) into the size of a lens array (CCTV SV-10035V). A sensor array (PointGrey Flea2-08S2C-C) was utilized for acquiring different sub-aperture images formed by corresponding imaging lenses. To introduce spatial-spectral coupled sampling, we inserted broadband colour filters with different spectral profiles between lens array and sensor array, as shown in Fig. 2a,b. 25 discretized beams after the image plane were selected to represent 25 plane waves for particular angles (particular spatial frequencies). These beams were further modulated by different colour filters. Then we applied a 4D deconvolution algorithm to the 25 high-resolution RGB images for the reconstruction of the hyperspectral volumetric information (4D data).

To fully utilize the redundancy of the spectral and spatial signal and enhance the light efficiency, the spectral profiles of the colour filter array should be broadband and as uncorrelated to each other as possible. However, it is hard and expensive to manufacture the colour filters with specific spectral responses and the spectral properties of interference filters change with different illumination angles. As a result, we selected the low-cost coloured glass filters as our absorptive colour filters. During all these kinds of coloured glasses, 25 glasses were selected, as their spectral responses are broadband and have few correlations as shown in Fig. 2c. With regard to the layout of the colour filter array, adjacent filters’ spectral responses were chosen to be as uncorrelated as possible (as shown in Fig. 2b), because more spectral information could be introduced with relatively small spatial variation. In addition, we selected the spectral response of the colour filter in the middle to be quite smooth along the spectral range (from 400 nm to 700 nm). The 25 cameras were synchronized via external hardware trigger and instantaneous high-throughput data for sufficient 5D sampling were acquired with master-slave server architecture^19^. The detailed parameters of optical components can be found in Supplementary Table S1.

### Validation of the proposed approach

We verified the effectiveness of our approach by simulating the forward imaging model, obtaining the measurements and comparing the reconstructed hyperspectral volumes with ground truths. To obtain the 4D hyperspectral volumetric data for simulation, the volumetric data of a brain section labelled with three different fluorescent dyes (Cy3, FITC and hoechst 33258) was captured sequentially by a confocal microscope (Olympus FV1000). Since we could get the accurate emission spectrum of each fluorescent dye, the hyperspectral volumetric data was synthesized as the ground truth with the assumption of the same excitation efficiency. The central spectral bands of these three fluorescent dyes are quite close, which makes it hard to separate different dyes with conventional filters due to the colour crosstalk. However, by linear spectral unmixing^24^ with ground truth hyperspectral profiles, these three kinds of dyes can be successfully separated. It usually takes more than 10 minutes for confocal microscopy to capture the 4D hyperspectral volumetric data with 512 × 512 × 30 pixels and 31 spectral channels. With optical parameters of our system, we simulated the snapshot images with broadband colour filters by projecting the 4D data according to equation (2). The simulated sensor images were then added with Poisson noise. We reconstructed the hyperspectral profile for each voxel in the volume by applying the iterative numerical solution in equation (6), and adopted the spectral unmixing approach to separate different dyes as shown in Supplementary Fig. S2 (Supplementary Movie 1). Red, green and blue volumes represent Cy3, FITC and hoechst 33258 dyes, respectively. It took about 10 minutes for the whole computation with an Intel Xeon CPU E3-1230 V2 (without GPU acceleration, 30 iterations). The visual comparisons of the reconstructed results with ground truth demonstrate that our approach achieves accurate hyperspectral volume reconstruction, which is comparable to confocal microscopy with spectral scanning systems.

**Figure 3:**
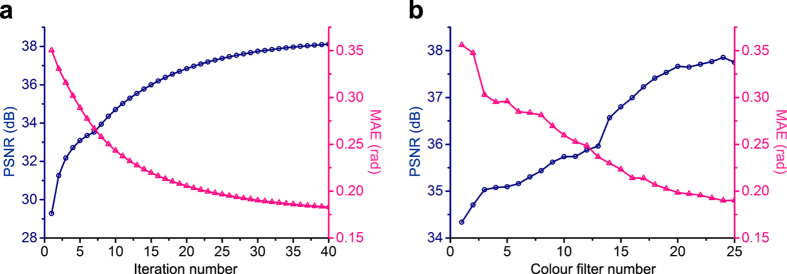
Quantitative analysis of numerical simulation. The influences from two key parameters—the number of iterations (**a**) and colour filters (**b**)—on the final 4D deconvolution performance, in terms of MAE and PSNR.

In order to quantitatively evaluate the reconstruction performance, we used two different evaluative criteria PSNR (peak signal to noise ratio) and MAE (mean angular error) for measuring the spatial and spectral accuracy, respectively. The MAE is calculated by averaging the cosine inverse of the inner product between the normalized ground truth and the reconstructed spectral vectors at different voxels^16^. Figure 3a shows the enhancing of PSNR and decreasing of MAE with the increasing of iteration number. The iteration will eventually converge to about 0.17 rad, which is quite good for hyperspectral sampling, i.e. 31 sampling points along the spectral range of 400 nm–700 nm at 10 nm interval, compared with other snapshot hyperspectral methods^16^. Therefore we set the iteration number to 30 in the final implementation corresponding to MAE of 0.18 rad and PSNR of 37.8 dB, which demonstrate the accuracy and robustness of the reconstructed results. In addition, we explore the influence of the broadband colour filter number on the reconstruction performance as shown in Fig. 3b. Since colour filters are used for spectral modulation and multiplexing, as expected, the result shows that the increase of the color filter number has much larger effect on the spectral accuracy than the spatial accuracy. As the final performance gradually converges with the increase of the colour filter number, we set the final number of the colour filters to 25, as shown in Fig. 1.

### Hyperspectral volumetric imaging in bright field

**Figure 4:**
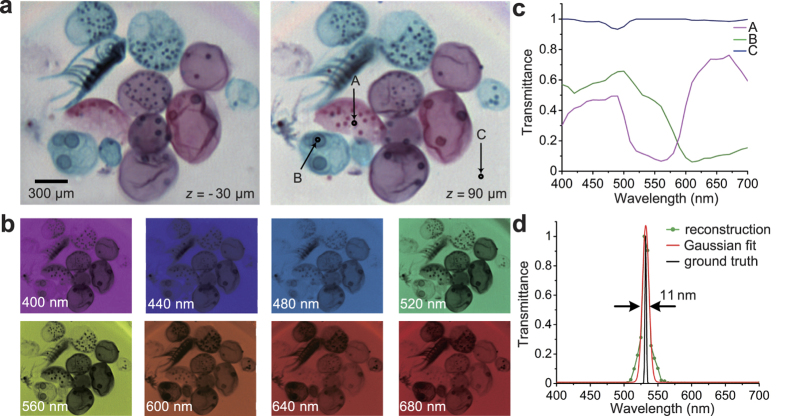
Hyperspectral volumetric imaging in bright field. (**a**) Different slices of the synthetic RGB volume for the volvox sample, by integrating the reconstructed hyperspectral data with colour response curves of PointGrey Flea2-08S2C-C. (**b**) Images of a selected depth (*z* = 30 *μm*) at several equally spaced spectrum channels for the volvox sample. (**c**) Spectrum profiles of the selected points in (**a**). (**d**) Comparison of the spectrum curves between ground truth (transmission data of the laser line filer with only 1nm FWHM) and the reconstruction with its gaussian fitted curve. FWHM, full width at half maximum measurement.

We first evaluated the capability of our system in bright field. The samples we used were the volvox, which is a genus of algae with various colours, and a laser line filter used to evaluate the spectral resolution. The 25 broadband colour filtered multi-view images of volvox were captured with our prototype system under bright field mode in a snapshot (exposure time is 30 ms). With the compressively-sampled input data (pixels number: 1024 × 768 × 3 × 25), the high-throughput hyperspectral volumetric data (voxels number: 617 × 528 × 30 × 31) can be reconstructed by the 4D deconvolution method proposed in this paper. In this experiment, we used a 10× air objective lens (Olympus, UPLSAPO10X2, N.A. = 0.4, F.N. = 26.5). The RGB slices at depths z = −30 *μm* and 90 *μm* shown in Fig. 4a were synthesized from the reconstructed hyperspectral images at corresponding depths. Figure 4b is the reconstructed hyperspectral images at depth *z* = 30 *μm*, and we show 8 spectra bands for illustration. The spectral profile of three different voxels marked in Fig. 4a (right) are shown in Fig. 4c. Different types of volvox can then be classified in 3D space based on the spectral information. This example demonstrates the effectiveness of our method to recover the transmission spectra of a semi-transparent volume within a snapshot. It provides us a powerful and convenient tool to do biological component analysis in real-time. Within a snapshot, we retrieved the information which traditionally takes lots of time to scan by commercial microscopic spectrum apparatuses (about one millisecond for only one voxel in the 3D space). Many applications such as 3D object recognition and accurate 3D segmentation can be applied and improved. Further comprehensive analysis for geology or cultural heritage can also be applied with the hyperspectral volumetric data^25^.

To further verify its ability to capture hyperspectral information, we imaged a laser line filter (Thorlabs FL532-1) with only 1 nm full width at half maximum measurement (FWHM). Figure 4d shows the comparison of the spectrum curves between the ground truth (transmission data of the laser line filer) and the reconstruction. The gaussian fitted curve (obtained by Levenberg-Marquardt algorithm) of the reconstructed spectrum is also shown in Fig. 4d to evaluate the spectral resolution. The FWHM of the gaussian fitted curve is about 11 nm. The center wavelength estimated by the reconstruction is about 532.12 nm, which also agrees well with the parameter of the laser line filter (532 ± 0.2 *nm*).

### Spectral unmixing of multi-fluorescence labelled immune tissue section

**Figure 5:**
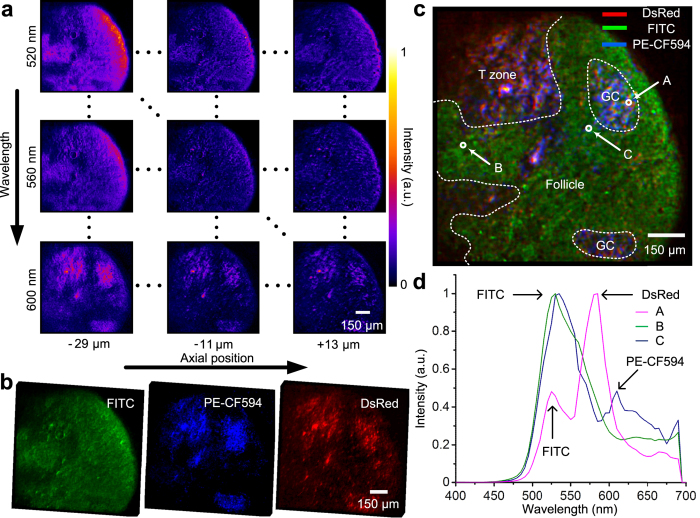
Experiments on a multi-fluorescence labelled lymph node sample. (**a**) Two-dimensional matrix of the images for the immune tissue at different wavelengths and axial positions. (**b**) The maximal intensity projections of the three unmixed fluorescence components from the reconstructed 4D data. (**c**) A selected slice of the unmixing result, rendered by three different colours in legend, and its manual anatomic zone labelling with dotted boundaries. (**d**) Reconstructed spectrum curves of the three selected points in (**c**). a.u., arbitrary units.

The missing spectral information largely restricts the current microscopic imaging approaches for distinguishing auto-fluorescence from labelled fluorescence and separating multiple fluorescence with overlapped spectrums. The typical example is the brainbow technology that randomly expresses different ratios of red, green, and blue derivatives of green fluorescent protein in individual neurons^23^. With multiple fluorescence involved, a more comprehensive understanding can be achieved. More spectral information may greatly help us to discriminate different fluorescence even though their spectral properties are quite similar and difficult to separate by colour filters. Moreover, the colour crosstalk can also be suppressed with the spectral information for fluorescence unmixing^24^. Therefore we demonstrate the capability of our method to analyze the multiple-fluorescence labelled biological structure of immune tissues. The sample is a lymph node labelled with three different fluorescence (FITC, PE-CF594 and DsRed). Our system was configured with a 20× air objective lens (Olympus, UPLSAPO20X, N.A. = 0.75, F.N. = 26.5) and a high pass emission filter (Olympus, U-FBW, cutoff frequency of 510 nm). We conducted snapshot fluorescence imaging of the sample with our system and reconstructed the hyperspectral volumes as shown in Fig. 5a. The linear spectral unmixing volumes of different fluorescent dyes are shown in Fig. 5b, which is the maximal intensity projection under a specific view. Our approach successfully eliminates the colour crosstalk introduced by the overlapped spectrums. Figure 5c shows the overlay of unmixing results at a depth z = 30 *μm*, and the spectral profiles (range from 400 nm to 700 nm at 5 nm step) of three individual voxels are demonstrated in Fig. 5d. In Fig. 5c, the anatomy of the lymph node can be divided into two parts. Inside is T zone enriched with T cells labelled by DsRed and outside is follicle enriched with B cells. IgD-FITC as the naive B cells marker was used to label follicle. Germinal center (GC) is the area of activated B cells labelled by CD95-PE-CF594. After immunization, GC could be seen in the center of follicle and T cells shown in red could migrate to GC.

### Imaging of dynamic fluorescence labelled samples

**Figure 6:**
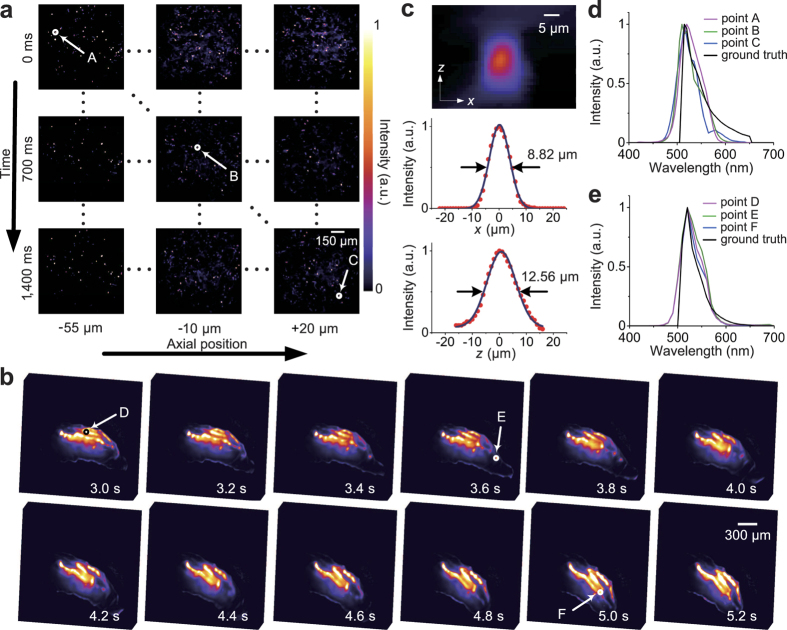
Observation of dynamic fluorescent beads and drosophila larvae. (**a**) Two-dimensional matrix of the images for fluorescent beads at different time instants and axial positions. (**b**) Time series of volumes for the GFP labelled drosophila larva (elav-Gal4/+; UAS-mCD8-GFP/+). (**c**) The reconstructed x–z plane of a fluorescent bead located at *z* = −10 *μm* showing the PSF, and corresponding x and z profiles to show lateral and axial resolution, respectively. (**d**) Comparison of the spectrum curves between ground truth (emission spectrum of the fluorescent beads) and the reconstruction at the selected points marked in (**a**). (**e**) Comparison of the spectrum curves between ground truth (emission spectrum of GFP) and the reconstruction at the selected points marked in (**b**). a.u., arbitrary units.

The proposed technology in this paper achieves hyperspectral volumetric recovery within a snapshot, which can be used for recovering dynamic multi-fluorescence labelled samples. To demonstrate this capability, we imaged the dynamic movement of fluorescent beads (F8811) and GFP labelled drosophila larvae (expressing membrane tagged GFP panneuronally), as shown in Fig. 6 and Supplementary Movies 2 and 3. The optical configuration in this experiment was the same to the experiment of spectral unmixing of multi-fluorescence labelled immune tissue. The reconstructed spectrum ranged from 400 nm to 700 nm at 5 nm step. Figure 6a shows multiple reconstructed hyperspectral volumes of fluorescent beads (Supplementary Movie 2). Each image of the matrix shows the moving fluorescent beads distributed at different axial positions and different time instants. Figure 6b demonstrates the hyperspectral volumetric video (frame rate of 5 fps) of a living drosophila larva (Supplementary Movie 3). The volumes were synthesized from the reconstructed hyperspectral volumetric data with spectral unmixing, and were shown as the maximum intensity projection along a specific view. Our hyperspectral volumetric video reveals the morphological changing of the translucent drosophila larva body and its neuron system during its movement. Since the size of the sub-diffraction fluorescent beads is only 200 nm, we can use it to evaluate the spatial resolution of the system by reconstructing the volume of a single bead^8^. Although the golden standard for spatial resolution in 3D space is to use two point targets separated by some specific distances^26^, it is hard to find such fluorescent samples, which should be arranged regularly in axial direction. Therefore, we used the same way described in the recent work^8^ to evaluate the spatial resolution. Figure 6c shows the reconstructed x-z plane of a fluorescent bead and the evaluation for spatial resolution (about 8.8 *μm* and 12.6 *μm* in the lateral and axial dimensions under FWHM, respectively). With the capability of capturing hyperspectral volumetric videos (5D data), lots of analysis, such as chromatography and fluorescence unmixing, can be done right after the specimen preparation. Even for static specimens, a waste of time for scanning and repetition can be avoided to greatly decrease the phototoxicity and raise the work efficiency. For each point of the 3D sample, we can get its spectral profile which may give us more opportunities to find new mechanisms or phenomena in both biology and materials science. The comparisons of the reconstructed spectral profile of fluorescent bead (F8811) and fluorescence (GFP) with the standard spectral profiles demonstrate the accuracy of the reconstructed hyperspectral volumes, as shown in Fig. 6d,e. The deviations between the peaks of the reconstructed fluorescence spectrum and the ground truth are all less than 11 nm.

## Discussion

Light field microscopy, which captures light field visual information (2D spatial and 2D angular dimensions), has been used merely for high-speed volumetric reconstruction of scattering or fluorescent samples. The spatial information of these samples is only 3D due to the isotropy of the emitted light from each 3D point^27^. Compared with traditional light field 3D deconvolution utilizing the redundant angular sampling, our 4D deconvolution algorithm trades off the enhanced spatial resolution for spectral acquisition. However, the system is easy to degrade to the light field microscopy by taking off the broadband colour filters, as shown in Supplementary Fig. S3. Then higher spatial resolution can be achieved for the applications without the need of spectral information. Analogously, for thin samples without axial information, the system can directly degrade to fast hyperspectral microscopy saving lots of computation time used for 4D deconvolution, see Supplementary Fig. S4. In this experiment, EGR1-EGFP and Thy1-EYFP^28^, whose spectral profiles are very similar with only 20 nm interval in central spectral bands, were separated by our method accurately. Their distributions in Supplementary Fig. S4 agree well with the descriptions in previous work^29^. This flexibility of the system is full of humanness, since many applications have their own emphasis and 4D data may not be necessary for all occasions.

The spatial resolution of the system can be further improved by many ways. Using a better objective lens with higher numerical aperture (NA) instead of the current one with 0.75 NA is the most immediate one. Furthermore the RGB sensors we used to provide more spectral sampling will decrease the spatial resolution due to its Bayer filter. In other words, the Bayer filter enlarges the pixel size of the sensor and decreases the final lateral resolution of the system by a factor of 2. Choosing a RGB sensor with a smaller pixel size or without the bayer filter (such as Foveon sensor) can indeed increase the lateral resolution up to the diffraction limit imposed by the objective lens.

In the future, we would like to explore new capabilities of the generalized multi-view microscopy for high-throughput imaging by replacing the broadband colour filter array with other optical elements such as polarizer or mask. Although broadband colour filters used in this paper can provide high-quality results, we are also exploring to optimize the spectral profiles of colour filters for better reconstruction performance. One possible way to improve the spectral resolution in the specific range is to use sharper interference filters, but these filters with multiple peak values are not widely available and expensive. In addition, more design choices, such as coded aperture and pixel interlaced sampling strategies, can be explored and extended for snapshot hyperspectral volumetric imaging with new algorithms and optical designs. We believe that the approach proposed in this paper will inspire the future computational microscopy design towards high-resolution and high-dimensional visual signal acquisition, and facilitate a wide range of applications.

Moreover, for most researchers in biology, the intervals of central emission spectrum bands for different fluorescence are usually chosen to be as far as possible in most biological applications to minimize the colour crosstalk. In the meantime, most fluorescent proteins will be excited together by two-photon excitation microscopy and their emission spectra are quite close, which further increases the difficulty of the discrimination for multiple fluorescence. SHVM provides a new effective and time-saving solution for this kind of applications. Without the concern about the similar spectral property of different dyes, we can break through the restrictions of limited choices for fluorescence. As a result, people can design new experiments which are unable or very difficult to realize with traditional techniques.

In summary, this paper presents a computational microscopic imaging approach that captures high-resolution and high-accuracy hyperspectral volumetric data at video rate. To the best of our knowledge, we are the first to realize hyperspectral volumetric video acquisition and demonstrate the great potential applications of our system. Compared with conventional temporal scanning methods, our approach combines optical design with computational reconstruction to fully utilize the sparsity of visual signal, which substantially reduces the acquisition time and avoids the photo-bleaching of fluorescent samples. A novel spatial-spectral coupled sampling system was designed to realize efficient multiplexing and the camera array was used to provide high-throughput acquisition. With the proposed 4D deconvolution reconstruction algorithm, we successfully retrieved the high-dimensional visual signal. We also validated our approach on different samples, which demonstrates the effectiveness of our approach and its various applications.

## Methods

### 4D point spread function (PSF)

If we remove the broadband colour filter array, our system is degenerated to light field microscopy. In the light field microscope, each view of the sample has a specific 3D point spread function which carries information about the 3D position of each voxel in the volume. Each image is the linear superposition integral of 3D volume with the corresponding 3D point spread function^27^. With 25 different broadband colour filters and the RGB Bayer colour filters in our array cameras, we create a 4D PSF (3D point spread function with spectral modulation) that carries both 3D spatial and 1D spectral information of each point in the volume. The spectral responses of the RGB Bayer filter of colour camera were calibrated by using a monochromoter with an integrating sphere and a photometer^30^, as shown in Supplementary Fig. S5. The 75 spatial-spectral coupled measurements captured at a single instant are the modulation of hyperspectral volumetric data with the spectral response of broadband colour filters and the RGB Bayer colour filter in spectral dimension and different 3D point spread functions in spatial dimension. The linear forward imaging model can be formulated as:


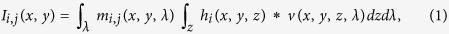


where * represents the convolution operator, *x*, *y* represents the spatial lateral dimension and *z* represents the axial dimension and *λ* represents the spectral dimension. *I*_*i*,*j*_(*x*, *y*) denotes the sensor image captured with camera *i*(*i* = 1, …, 25) and RGB colour channel *j*(*j* = 1, …, 3); *m*_*i*,*j*_(*x*, *y*, *λ*) represents the spectral modulation introduced by the *i*th broadband colour filter combined with the *j*th RGB Bayer colour filter; *h*_*i*_(*x*, *y*, *z*) is the 3D PSF of the *i*th camera and *v*(*x*, *y*, *z*, *λ*) is the 4D hyperspectral volumetric images.

### 4D deconvolution algorithm

In conventional 3D deconvolution algorithms^27^,^31^, multiple aliased and low-resolution images, such as multi-view images or focal stack, of semi-transparent objects and fluorescence labelled samples can be computationally combined to recover volumetric images with higher resolution. We make use of this extra spatial information for spectral acquisition. As fluorescence is incoherent in both spatial and temporal dimension^32^, the spectral distribution of the fluorescence for each slice of the reconstructed volume should be the same for different angles of light. Since the unknown hyperspectral volumetric images have intrinsic redundancy in both spatial and spectral dimensions, the proposed 4D deconvolution algorithm uses the expectation maximization (EM) method^33^ along with the total variation (TV) regularization^34^ to solve the underdetermined system in equation (1). In practice, equation (1) is discretized as:


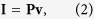


where **I** ∈ **R**^**M**^ and **v** ∈ **R**^**N**^ are the vectorized camera array measurements and vectorized hyperspectral volumetric data. **P** ∈ **R**^*M*×*N*^ is the projection matrix determined by *h*(*x*, *y*, *z*) and *m*(*x*, *y*, *λ*) in equation (1). We are seeking to solve the inverse problem of equation (2), i.e. given the spatial-spectral coupled measurements recorded by the sensor array, and estimate the spectral profile at each point in the volume. This process is equivalent to the 4D tomography problem^17^,^35^. Since the spectral modulation is uniform for each voxel, it will not influence the periodicity of the projection matrix. This provides us the opportunity to greatly reduce the computational burden of the forward projection and backward projection by convolution.

In our implementation, we assume the imaging noise follows Poisson distribution^27^, and the background noise is calibrated by subtracting the captured images with pre-captured background images. Then the 4D deconvolution algorithm can be formulated as the following optimization:


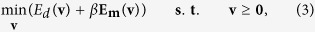


where *β* (usually *β* = 1) is the coefficient that balances the TV regularization term *E*_*m*_(**v**) and data term *E*_*d*_(**v**). The data term is defined as:


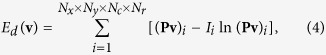


where ln (·) represents the logarithm operator; *N*_*x*_ and *N*_*y*_ represent the 2D spatial resolution of each camera; *N*_*c*_ and *N*_*r*_ represent the numbers of cameras and RGB Bayer colour filters. For the TV regularization term, we incorporate the sparse prior of natural fluorescent samples to utilize the piecewise smoothness of the reconstructed hyperspectral volume:


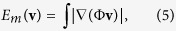


where 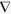 is the spatial gradient and Φ is the spectral projection. By utilizing the Karush-Kuhn-Tucker (KKT) condition and the complementary slackness condition^34^, the numerical solution of equation (3) is


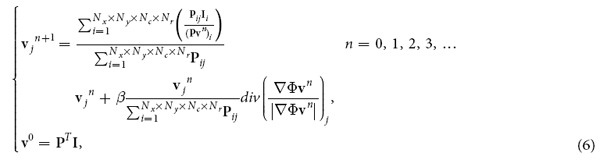


where 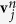 represents the *j*th element of the vectorized 4D data **v** in *n*th iteration. We repeat the iterative updating rules in equation (6) until it converges, or *n* attains the predefined maximum iteration number *n*_max_ to obtain the hyperspectral volumetric data.

### Calibration

For geometrical calibration, we put a checkerboard on the image plane at the export of the microscope. All cameras were focused on the checkerboard by adjusting the focusing position of the lens one by one. At least eight corner points distributed all around the checkerboard were chosen to be the reference points used for homography mapping from different views to the center view.

For accurate spectral retrieval from the multiplexed data, the spectral response of the whole system should be measured precisely. We first got the spectral responses of the microscope and the lenses from their data sheets. The RGB responses of the sensors were measured one by one, using a monochromator and an integrating sphere (Supplementary Fig. S5). A photometer was also used to measure the actual intensity of the monochromator. For bright field imaging, different band-bass filters with known spectral profiles were placed at the image plane to calibrate the spectral response of the illumination.

The ground truth in Fig. 6b,d was created by the emission spectra of the fluorescent samples and the spectral response of the filters in microscopy. The emission spectra of the fluorescent beads (Carboxylate-Modified Microspheres with yellow-green fluorescent, F8811) and GFP can also be found from the websites of Thermo Fisher^36^. And the fluorescence mirror unit of the microscope we used for these experiments is U-FBW (Olympus). Its high pass emission filter has great suppression for the spectrum below 520 nm, for which we assume 90% at 515 nm, 50% at 510 nm and 0% below 510 nm.

### Preparation for immune tissue section

C57BL/6 mice were transferred with DsRed-labelled T cells and immunized with antigen so that red T cells could be seen in germinal center (GC) area. Mice were sacrificed on day 5 post immunization and lymph nodes were harvested and fixed with 0.05 M phosphate buffer containing 0.1 M L-lysine and 1% paraformaldehyde at 4 °C. They were dehydrated in 30% sucrose solution over night. Tissues were stored at −80 °C. 70 *μm* sections were then made with Leica CM1950. Sections were blocked for 1 hour with a 1% bovine serum albumin block solution containing 0.3% Triton X-100, and then stained with IgD-FITC and CD95-PE-CF594 to label follicle and GC area, respectively. All animal experiments were performed in accordance with the Institutional guidelines of Tsinghua University and were approved by the Animal Care and Use Committee at Tsinghua University.

### Drosophila larvae

All flies used in this study were raised on standard cornmeal medium. elav-GAL4 and UAS-mCD8-GFP lines were crossed to generate flies expressing membrane tagged GFP panneuronally. Third instar larvae were used for imaging. To restrict the larva from moving out of the field of view, it was pinned on the cover glass.

## Additional Information

**How to cite this article**: Wu, J. *et al.* Snapshot Hyperspectral Volumetric Microscopy. *Sci. Rep.* **6**, 24624; doi: 10.1038/srep24624 (2016).

## Supplementary Material

Supplementary Information

Supplementary Movie 1

Supplementary Movie 2

Supplementary Movie 3
