# Supplementary material for: Snapshot Hyperspectral Volumetric Microscopy
Source: Sci Rep. 2016 Apr 22;6:24624. doi: 10.1038/srep24624 (PMC4840377; doi:10.1038/srep24624)
Supplement: Supplementary Information [file srep24624-s1.pdf]

Supplementary Information

Snapshot Hyperspectral Volumetric Microscopy

Jiamin Wu, Bo Xiong, Xing Lin, Jijun He, Jinli Suo, and Qionghai Dai

Department of Automation, Tsinghua National Laboratory for Information Science and Technology (TNList), Tsinghua University, Beijing, 100084, China

**Supplementary Figure S1**	Illustration of the prototype system
**Supplementary Figure S2**	Simulation results of fluorescence unmixing based on the reconstructed hyperspectral volumetric data
**Supplementary Figure S3**	Comparison between digital refocusing and 3D deconvolution with the degenerating of our approach to camera array based light field microscopy
**Supplementary Figure S4**	Degrading to hyperspectral microscopy for fluorescence unmixing
**Supplementary Figure S5**	The method for calibrating the spectral response of RGB sensors
**Supplementary Table S1**	Optical parameters of the used components
**Supplementary Video 1**	Numerical simulations for quantitative evaluation of the proposed approach
**Supplementary Video 2**	The hyperspectral volumetric video of dynamic fluorescent beads
**Supplementary Video 3**	The hyperspectral volumetric video of GFP labeled drosophila larval

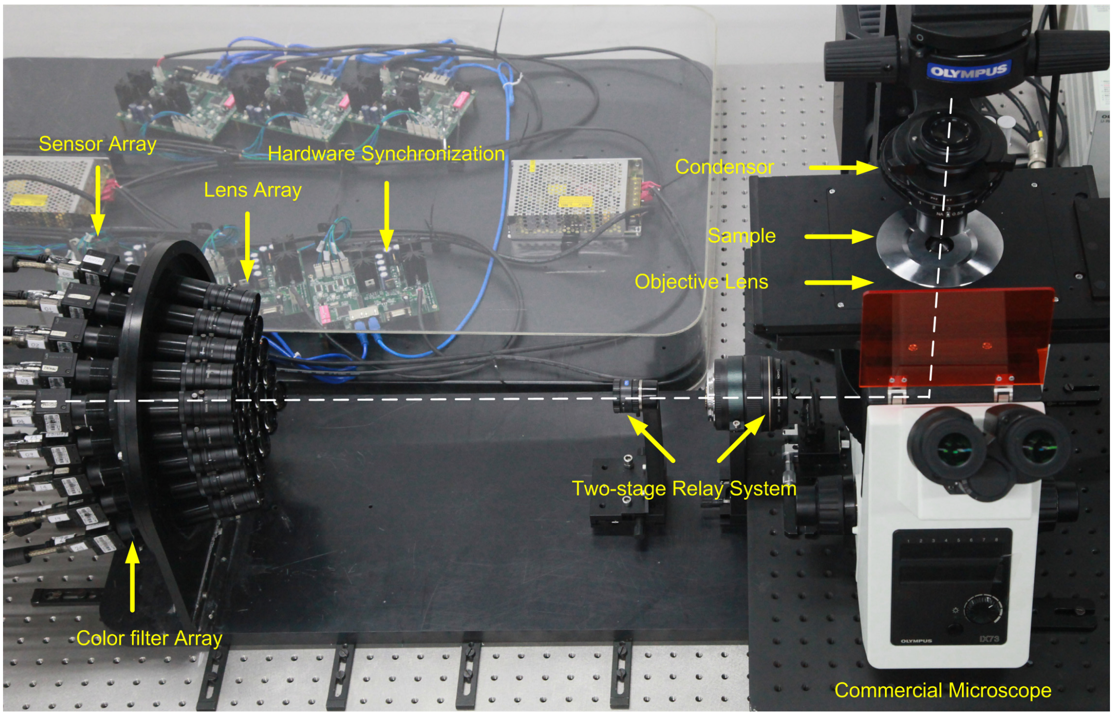

Supplementary Figure S1: **Illustration of the prototype system.** Photograph showing the prototype system of snapshot hyperspectral volumetric microscopy (SHVM) that using array cameras along with broadband color filters for providing effective multiplexing of visual signal and high throughput data acquisition of microscopic samples.

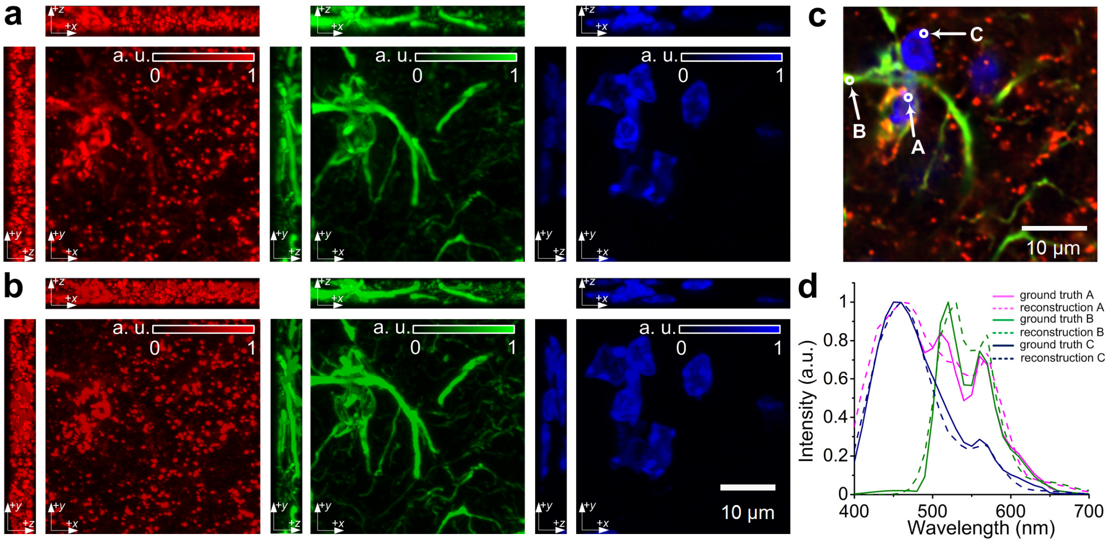

Supplementary Figure S2: **Simulation results of fluorescence unmixing based on the reconstructed hyperspectral volumetric data.** (**a**) Our unmixed components corresponding to three dyes. (**b**) Ground truth counterparts for comparison. (**c**) RGB rendered unmixing result at a selected depth (z-plane), with three color channels corresponding to three different dyes. (**d**) Comparison between the reconstructed spectrum curves of three points in c and their ground truth version. a.u., arbitrary units.

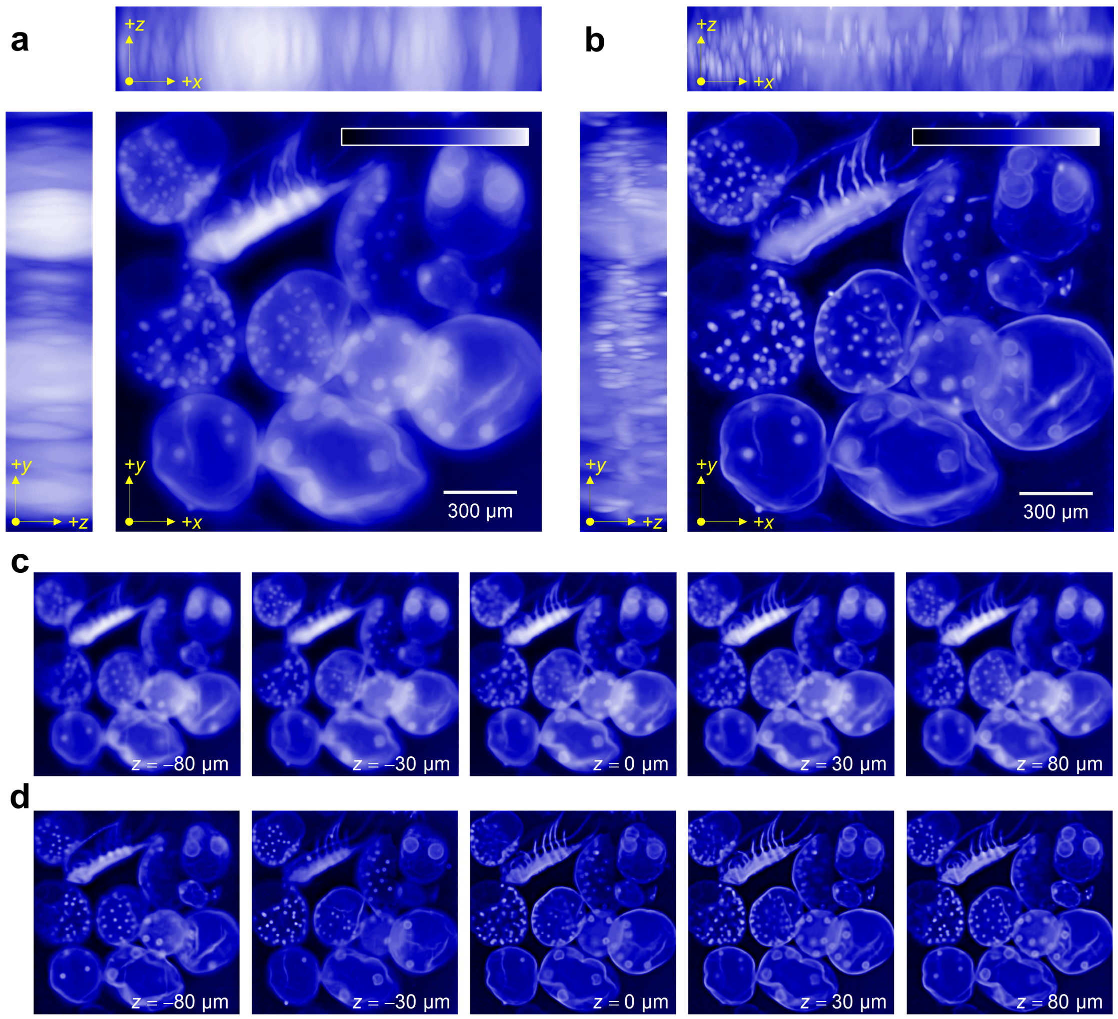

Supplementary Figure S3: **Comparison between digital refocusing and 3D deconvolution with the degenerating of our approach to camera array based light field microscopy.** By removing the color filters inserted between the lens array and sensor array, the snapshot hyperspectral volumetric imaging system can be degraded to camera array based light field microscopy to achieve enhanced spatial resolution introduced by 3D volumetric deconvolution without spectral information. (**a**) Maximum intensity projections of the volvox volume reconstructed by digital refocusing. (**b**) Maximum intensity projections of the volume of the volvox reconstructed by 3D deconvolution with higher spatial and axial resolution. (**c**) Slices obtained by digital refocusing. (**d**) Corresponding slices obtained by 3D volumetric deconvolution. Compared with the focal stack synthesized by digital refocusing of the light field, 3D deconvolution indeed enhances the optical section and spatial resolution of the volumetric data.

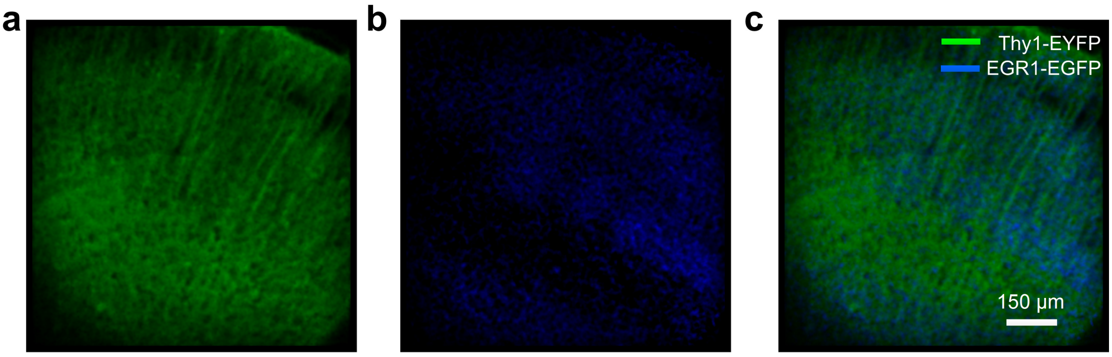

Supplementary Figure S4: **Degrading to hyperspectral microscopy for fluorescence unmixing.** Thin samples have little axial information, so the 4D hyperspectral volumes degenerate to 3D hyperspectral images. And the images captured by different cameras in our approach correspond to the same plane of the sample without containing any depth information. A much quicker 3D hyperspectral deconvolution algorithm can be easily applied to the captured data for reconstructing hyperspectral images within a snapshot. We demonstrated an experiment on the coronal slice of Thy1-EYFP / EGR1-EGFP double transgenic mice to separate the EYFP and EGFP, whose spectral profiles are very similar with only 20nm interval in central spectral bands and hard to be distinguished by conventional filters. Here is the result of fluorescence unmixing with the reconstructed hyperspectral images. Their distributions agree quite well with previous work. (**a**) Separated Thy1-EYFP result in cortex layer 5 neurons soma and apical dendrites. (**b**) Separated EGR1-EGFP result. EGFP was expressed in cortex neurons: especially in layer 4 and layer 6 while layer 2, 3 and 5 show a sparse expression. (**c**) Overlay of these two channels.

For slice preparation: Thy1-EYFP / EGR1-EGFP double transgenic mice were generated from crossing between Thy1-ChR2-EYFP and EGR1-EGFP. Mice were overdosed with 400ul 2% phenobarbital sodium and perfused transcardially with cold Phosphate Buffered Saline (PBS), followed by 4% paraformaldehyde (PFA) in PBS. Brains were extracted from the skulls and kept in 4% PFA at 4 °C overnight, then transferred to 20% sucrose in PBS. 10/50 μm coronal slices were taken using a vibratome and collected in cold PBS. Finally we added some Antifade Mounting Medium and put coverslipping on microscope slides. All animal experiments were performed in accordance with the Institutional guidelines of Tsinghua University and were approved by the Animal Care and Use Committee at Tsinghua University.

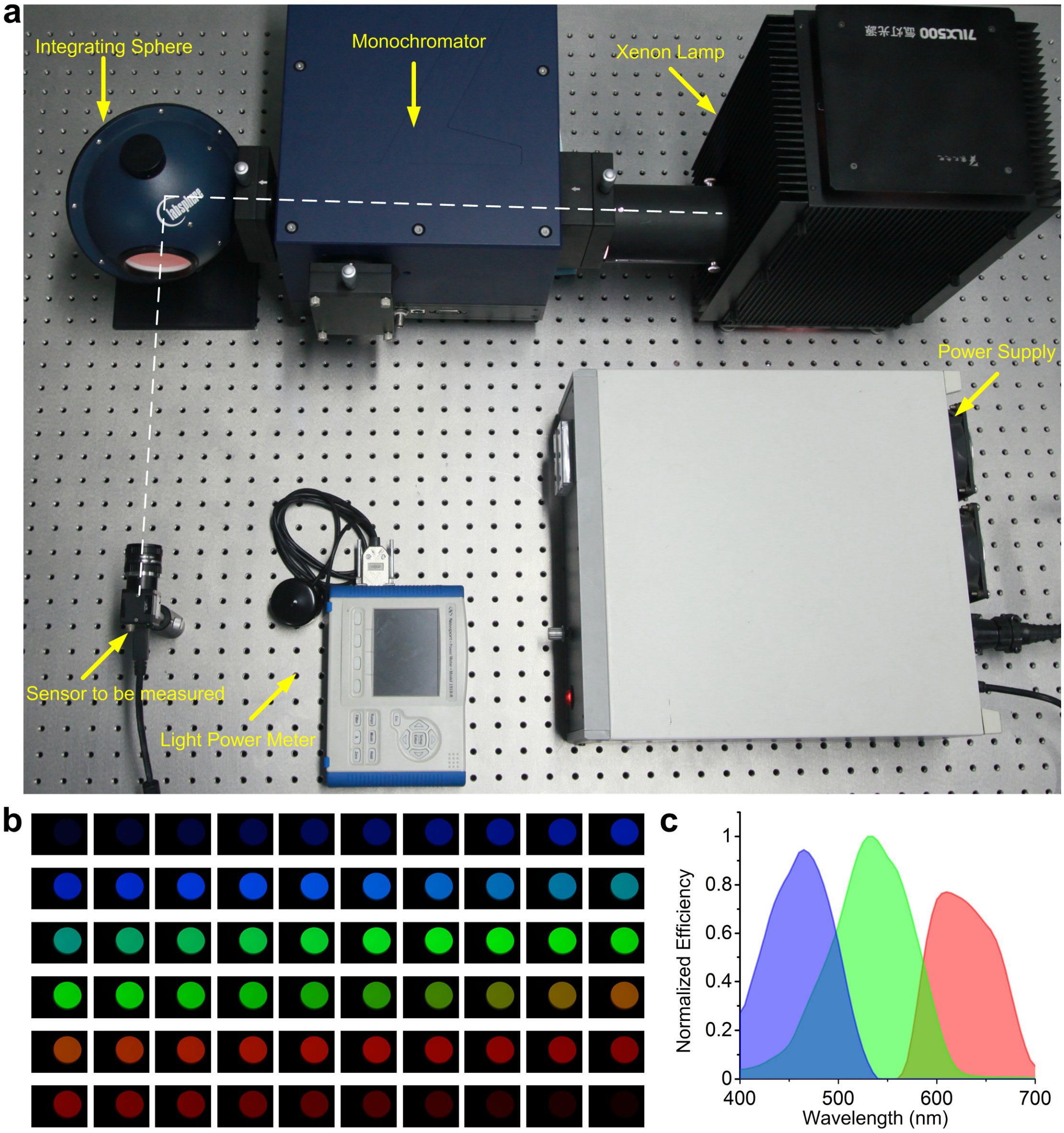

Supplementary Figure S5: **The method for calibrating the spectral response of RGB sensors.** (**a**) Photograph showing the device used to measure the spectral response of 25 RGB sensors (PointGrey Flea2-08S2C-C) under sensor parameter settings the same as real-capture experiments. A xenon lamp and a monochromator were used to provide monochromatic illumination. An integrating sphere was connected with the export of the monochromator to make the illumination uniform. Each measuring sensor from 25 sensors was set the same parameters as real-capture experiments and placed at the same place to capture the outgoing light of the integrating sphere. A light power meter was used to get the reference of the illumination intensity when different spectral bands were chosen by the monochromator. With the images captured by the sensor under different spectral bands (**b**) and the reference intensity obtained by light power meter, the RGB response of the camera (**c**) was calculated.

Supplementary Table S1: **Optical parameters of the used components**

Supplementary Fig.1	Type	f in mm	$f/\#$	Manufacturer
Commercial Microscope	IX73			Olympus
Objective Lens	UPLSAPO 20X, NA 0.75, W.D. 0.6 mm			Olympus
	UPLSAPO 10X2, NA 0.4, W.D. 3.1 mm			Olympus
Two-stage Relay System	Canon EF	85	1.8	Canon
	Lens M0814-MP2	8	1.4	Computar
Lens Array	CCTV Lens SV-10035V	100	3.5	VS Technology
Color Filter Array	CB2, JB9, LB3, LB6, LB7, LB9, LB10, LB11, LB12, LB16, LB17, LB18, PNB586, QB1, QB2, QB9, QB10, QB12, QB19, QB21, QB23, QB26, QB29, SJB130, SSB130			Nantong Yinxing Optical Glass
Sensor Array	Flea2-08S2C-C, 1024×768, RGB Sensor			Point Grey
